# A Robust Static Headspace GC-FID Method to Detect and Quantify Formaldehyde Impurity in Pharmaceutical Excipients

**DOI:** 10.1155/2018/4526396

**Published:** 2018-03-04

**Authors:** Bashir Daoud Agha Dit Daoudy, Mohammad Ammar Al-Khayat, Francois Karabet, Mohammad Amer Al-Mardini

**Affiliations:** ^1^Department of Pharmaceutical Chemistry and Quality Control, Faculty of Pharmacy, Damascus University, Damascus, Syria; ^2^Department of Chemistry, Faculty of Science, Damascus University, Damascus, Syria

## Abstract

Formaldehyde is a highly reactive impurity that can be found in many pharmaceutical excipients. Trace levels of this impurity may affect drug product stability, safety, efficacy, and performance. A static headspace gas chromatographic method was developed and validated to determine formaldehyde in pharmaceutical excipients after an effective derivatization procedure using acidified ethanol. Diethoxymethane, the derivative of formaldehyde, was then directly analyzed by GC-FID. Despite the simplicity of the developed method, however, it is characterized by its specificity, accuracy, and precision. The limits of detection and quantification of formaldehyde in the samples were of 2.44 and 8.12 *µ*g/g, respectively. This method is characterized by using simple and economic GC-FID technique instead of MS detection, and it is successfully used to analyze formaldehyde in commonly used pharmaceutical excipients.

## 1. Introduction

Although considered pharmacologically nonactive, pharmaceutical excipients have critical effects on drug product safety, efficacy, and quality. During early stages of drug formulation development, excipients are evaluated for appropriateness by studying their physical and chemical compatibility with drug substances under stressed and accelerated conditions. Depending on the results of the compatibility studies, formulator can exclude incompatible excipients from later stages of formulation development. Chemical incompatibility with drug substances may result not only from direct reaction with excipients but also from reaction with excipient impurities. These impurities include formaldehyde which could be generated by autoxidation degradation of many pharmaceutical excipients such as polyvinylpyrrolidone (PVP) and polyethylene glycol (PEG) [1–4].

Pharmacopoeial monographs of pharmaceutical excipients rarely require testing for formaldehyde specifically [5, 6]. However, formaldehyde may negatively affect safety, efficacy, chemical stability, and performance of dosage forms, even in trace amounts. It can form adducts with many pharmaceuticals containing nucleophilic functional groups, especially amine and hydroxyl groups [1, 2, 4], such as gelatin [7], varenicline [8], irbesartan [9], phenylephrine [10], and GDC-0973 [11]. Some of these adducts may have toxic effects such as the degradation product of melatonine with formaldehyde [12].

Formaldehyde has a low molecular weight and a high chemical reactivity. Furthermore, it has little UV activity and low detector sensitivity and specificity. Besides, it is soluble in both water and organic solvents. As a consequence, it is difficult to directly extract and determine formaldehyde in a specific, accurate, and sensitive way. Therefore, most of the analytical methods used for formaldehyde analysis employ chemical derivatization to improve its stability and modify the physicochemical properties to enhance its detectability [11, 13–17].

Many analytical methods have been developed to determine formaldehyde in pharmaceuticals and cosmetics. These methods are based on colorimetric, spectrophotometric, fluorescent, capillary electrophoresis, HPLC, GC, and GC-MS techniques [1, 17–20]. Lots of the reported methods have relied on derivatizing formaldehyde using acetylacetone or chromotropic acid reagents prior to colorimetric and/or spectrophotometric determination. However, the main disadvantages of these methods are the lack of appropriate specificity and/or sensitivity [1, 17, 20, 21]. 2,4-Dinitrophenylhydrazine, the most popular derivatization reagent, has been used before determination of formaldehyde by HPLC [11, 15, 22]. However, some of these methods have limited selectivity or have suffered from difficulty handling and injecting excipient samples which form viscous solution (such as PVP). In addition, other HPLC methods require tedious and multisteps extraction procedures and/or long analysis time [1, 16, 20, 23]. Gas chromatography is the best choice for determination of volatile components, especially when associated with the headspace sampling technique. This technique provides a simple way to directly inject the extracted volatiles into GC. In contrast with the direct injection of the sample solution into GC inlet, the headspace injection allows the volatiles to be analyzed without interference by the nonvolatile matrix. By using the headspace technique, analysts can also simply derivatize target components by utilizing the headspace vial as a reaction vessel [24]. Few GC methods have been developed to determine formaldehyde in pharmaceutical excipients; however, the derivatization reaction, the extraction, and/or the sample preparation were generally complex and/or required long time [13, 16, 19, 20]. del Barrio et al. reported a headspace GC-MS method for determination of formaldehyde in some excipients by converting it to diethoxymethane using acidified ethanol. In this method, the sample preparation was very simple and rapid, and the chemical derivatization can be carried out under mild conditions. However, the authors did not evaluate the capability of using FID for quantification [16]. Although FID is less sensitive than MSD, it is the most widely used detector for routine quantification in typical analytical laboratories. In addition, it is easier to operate and maintain [20, 25].

As most of the spectrophotometric methods have some disadvantages, while other chromatographic methods are complex to be performed, the aim of this research is to develop and validate a static headspace (SHS) GC-FID method to determine formaldehyde, and then to use it as a screening method and a quality control tool to analyze this impurity in commonly used pharmaceutical excipients.

## 2. Materials and Methods

### 2.1. Standards, Reagents, and Chemicals

High-purity diethoxymethane (≥99.0%) was purchased from Aldrich (USA). Absolute ethanol (99.9%) and pure formic acid were purchased from Panreac (Spain). Formaldehyde solution (37–41%) was obtained from SCP (England). ACS grade *p*-toluenesulfonic acid monohydrate (≥98.5%) was purchased from Sigma-Aldrich (Japan). All excipients used were of pharmaceutical grade.

### 2.2. Instrumentation

Experiments were conducted by Agilent Model 7890A gas chromatograph equipped with mass selective detector (MSD model 5975C) and associated with GC sampler 80 enhanced with Agilent PAL headspace option. Agilent MSD Productivity ChemStation E.02.01.1177 software was used to control, acquire, and process the chromatographic data. A 30 m × 0.25 mm i.d. ZB-WAX column with 0.25 *µ*m film thickness (Phenomenex, USA) was utilized for gas chromatographic separation.

### 2.3. Headspace Sampling Parameters

The headspace autosampler parameters were set as follows: incubation temperature: 70°C; incubation time: 25 min for PVP samples and 15 min for PEG samples; syringe temperature: 75°C; agitation speed: 500 rpm; syringe injection volume: 800 *µ*l; syringe fill speed: 100 *µ*l/s; syringe injection speed: 1000 *µ*l/s; fill strokes: 1; pull-up delay: 2 s; pre- and postinjection delay: 0.2 and 0.4 s, respectively. Carryover in the headspace syringe was eliminated by an automatic syringe flush performed after each injection.

### 2.4. GC Instrumental Conditions

The injector was maintained at 170°C with a split ratio of 1 : 25. The column oven temperature program involved an initial temperature of 35°C for 5 min and increased at 40°C/min to 220°C and held for 1 min. The carrier gas was helium (99.999%) at a constant flow rate of 0.9 mL/min. FID was set at 280°C for quantification. MS detection was carried out at 230°C with full scan (31–250 amu) for identification.

### 2.5. Sample Preparation

Samples were prepared by directly weighing 250 mg of the tested excipient into a 20 ml amber headspace vial (Supelco). After that, 5 mL of the solution of 1% (w/w) *p*-toluenesulfonic acid in ethanol was added to the content of each sample vial which was then immediately sealed with a magnetic screw cap lined with a butyl/polytetrafluoroethylene septum (Supelco) and shaken for 2 minutes (until the content becomes completely dissolved). Finally, the prepared vials were sequenced and automatically moved to the incubator for completing the chemical derivatization of formaldehyde.

### 2.6. Standards Preparation

The concentration of formaldehyde solution was determined by applying the iodometric method described in British Pharmacopoeia 2013 [5]. The concentration was 35.10% (w/w).

Standard solutions of formaldehyde were prepared in the acidified ethanol (1% *p*-toluenesulfonic acid). A stock standard solution of formaldehyde at 1251.063 *µ*g/ml was prepared and used to prepare a series of standard solutions at lower concentrations by serial dilutions. Then, 5 mL of each standard solution was transferred to a headspace vial and treated as described above.

### 2.7. Method Validation

The method was validated in terms of specify, linearity, accuracy, repeatability, intermediate precision, limit of detection, and limit of quantification according to British Pharmacopoeia 2013 [5].

### 2.8. Statistical Analysis

Results were statistically processed by *t*-test or one-way analysis of variance (ANOVA) in order to evaluate significant differences (*p* < 0.05). Statistical Package for the Social Sciences (SPSS, 20) software was used for this purpose.

## 3. Results and Discussion

### 3.1. Development and Optimization of SHS-GC-FID Method

#### 3.1.1. Chromatographic Conditions

**Figure 1:**
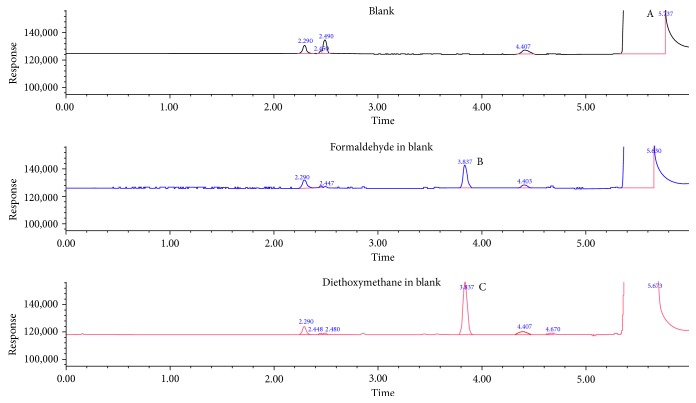
Overlaid chromatograms show the conversion of formaldehyde in acidified ethanol to the corresponding derivative. (A) Ethanol; (B) formaldehyde derivative; (C) diethoxymethane standard.

*(1) Identification*. The identification of the derivative, the product of the chemical reaction of formaldehyde with ethanol, was confirmed by using the corresponding standard (diethoxymethane). As shown in Figure 1(B), the retention time of formaldehyde derivative peak (*t*_*R*_ = 3.837 min) matched with that belonging to the corresponding standard peak eluted according to Figure 1(C). This indicates that the derivative was diethoxymethane.

**Figure 2:**
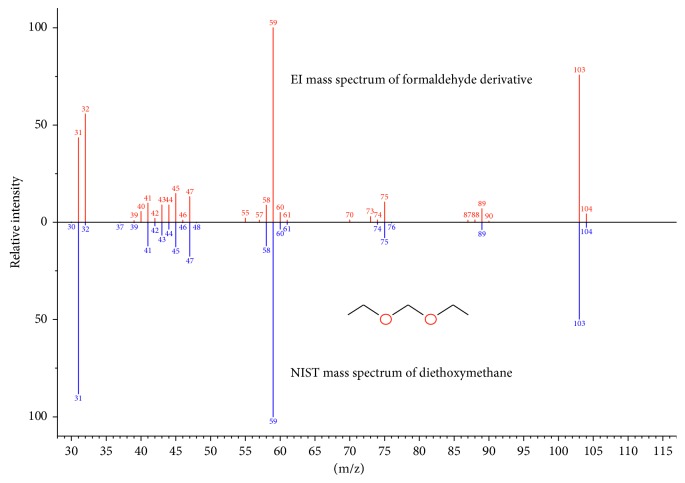
EI mass spectrum of formaldehyde derivative.

To further confirm the identity of formaldehyde derivative, EI mass spectrum of the corresponding peak (shown in Figure 2) in full scan was acquired and identified using the NIST MS library as diethoxymethane.

#### 3.1.2. Optimization of Sample Preparation and Headspace Sampling

*(1) Preliminary Investigation*. At the beginning, the sample preparation and the headspace sampling parameters described by del Barrio et al. [16] (the sample concentration: 100 mg/mL; the headspace parameter: incubation at 60°C for 15 min) were applied for analyzing and evaluating the recovery of formaldehyde derivative from the spiked samples of PEG 400 and PVP K-30. The results (*n* = 2) demonstrated that the mean recovery of formaldehyde derivative from PEG 400 was within the acceptable range (80–120%); however, it was not so for PVP K-30 (56%).

**Figure 3:**
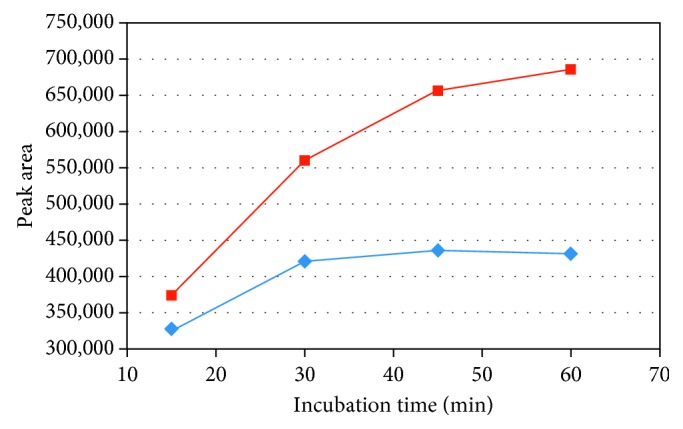
Effect of incubation time at 60°C on formaldehyde derivative peak area in PVP K-30 when the sample concentration is 50 mg/mL (blue box) and 100 mg/mL (red box).

In order to explore the factors that leaded to such low recovery of formaldehyde derivative from PVP K-30, the sample matrix effect and the previous incubation parameters were checked. First, the experiments carried out on the ethanolic solution of diethoxymethane standard showed that the incubation time of 15 min at the temperature of 60°C was sufficient for diethoxymethane to reach the static headspace equilibrium. Second, the mean recovery of diethoxymethane standard was 110% (*n* = 2) when preparing PVP K-30 samples by adding 5 mL of the ethanolic standard solution. Thus, there is no negative effect of PVP K-30 sample matrix on formaldehyde derivative extraction. Third, the incubation time required to complete the derivatization reaction of formaldehyde in PVP K-30 was evaluated at the temperature of 60°C. As shown in Figure 3, the incubation for 15 min was not sufficient for completing the derivatization reaction of formaldehyde. Rather, the incubation time should be adjusted at 45 min at least to ensure completion of the reaction. This difference in the incubation times required for PVP K-30 sample may be due to existence of a relationship between the physical nature of the sample and the kinetic of the derivatization reaction of formaldehyde.

*(2) Effect of Sample Dilution on Incubation Time*. The relatively high viscosity of ethanolic solution of PVP K-30, when prepared at concentration of 100 mg/mL, may be the reason behind the long time required to reach equilibrium for formaldehyde derivative (Figure 3). Although sample dilution can decrease sensitivity of the analytical method, however, it can reduce viscosity of the ethanolic solution of PVP K-30, which can in turn decrease the required time for both completing the derivatization reaction and reaching equilibrium for formaldehyde derivative between the sample and the headspace gas phases [24]. Therefore, the sample concentration of 50 mg/mL was used to evaluate the required incubation time for the spiked PVP K-30 samples while fixing the incubation temperature at 60°C. Figure 3 shows the diagram which correlates between the peak area of the derivative and the increased incubation time. The results demonstrated that there was no significant increase in the peak area of formaldehyde derivative after 30 min incubation.

As a consequence, sample dilution from 100 mg/mL to 50 mg/mL gave the advantage of lowering the incubation time to 30 min (instead of 45 min at least). Therefore, the sample concentration of 50 mg/mL was selected to complete the other experiments.

**Figure 4:**
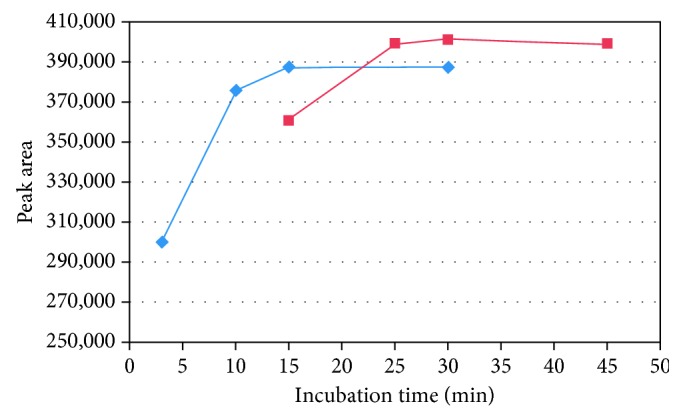
Effect of incubation time at 70°C on formaldehyde derivative peak area. Formaldehyde in PVP K-30 (red box) and formaldehyde in PEG 400 (blue box).

*(3) Effect of Increasing the Incubation Temperature*. Increasing the incubation temperature generally leads to an increase in the rate of the derivatization reaction and the headspace sensitivity. On the other hand, it results in decreasing the required time to reach the static headspace equilibrium [24]. Therefore, the incubation temperature was raised to 70°C (higher temperatures were not experimented to avoid any potential degradation of the samples). The results (shown in Table 1) indicate that there was no significant increase (*t*-test, *p* > 0.05) in the detector response when the reaction mixture was incubated at 70°C (instead of 60°C) for 30 min. Hence, there was no increase in the sensitivity. In contrast, the incubation time required to complete the derivatization reaction of formaldehyde in excipient samples decreased.  Figure 4 shows the plateau of the peak area of formaldehyde derivative with increase in incubation time at 70°C for both PEG 400 and PVP K-30. The data show no significant increase in the peak area of formaldehyde derivative after 15 min for PEG 400 and 25 min for PVP K-30.

As a consequence, the increasing of incubation temperature from 60°C to 70°C leaded to a decrease in the incubation time from more than 15 min to 15 min for PEG 400 and from 30 min to 25 min for PVP K-30. Therefore, the incubation temperature of 70°C was selected.

*(4) Headspace Sampling Parameters*. Headspace sampling parameters other than incubation time (injection speed and injection volume) were optimized and set in a way that can achieve a balance between the sensitivity and the peak shape parameters. The final headspace sampling parameters are given in Section 2.3.

#### 3.1.3. Effect of Water Presence on Formaldehyde Determination

Diethoxymethane formation reaction is susceptible to water presence which can reverse the reaction [16, 26]. Therefore, the tolerance of the derivatization reaction of formaldehyde to water was evaluated by adding it to several vials containing 5 mL of the reaction mixture (formaldehyde in acidified ethanol at a concentration of 1.5 *µ*g/mL) at the following amounts: 0, 12.5, 25, and 50 mg (0, 5, 10, and 20% w/w relative to the sample weight 250 mg). After analysis and statistical processing, the results (shown in Table 2) manifested that there was no significant difference (ANOVA test,*p* > 0.05) between the control (0% water) and the other samples.

As a consequence, excipient samples containing water ≤ 20% (w/w) can be analyzed without any significant effects on formaldehyde determination. Higher amounts of water were not experimented because the acceptable limits of water content stated in the monographs of PEG and PVP are 1–2% and 5%, respectively [5, 6].

### 3.2. SHS-GC-FID Method Validation

#### 3.2.1. Specificity

The specificity of the method was determined by comparing the chromatograms (Supplementary Material Figure S1) of the blank (acidified ethanol), the stressed excipients dissolved in ethanol and the standard solution of formaldehyde in the blank. The chromatograms showed that there were no interferences at the retention time of formaldehyde derivative peak. In addition, all volatile component peaks, which appeared after analyzing spiked excipient samples, dissolved in the blank (Supplementary Materials Figures S2 and S3) were completely separated from the analyte peak (the resolution between the analyte peak and any other one was more than 2).

#### 3.2.2. Linearity

The linearity of the method was evaluated from injection of nine concentrations of formaldehyde at the range of 0.25–50 *µ*g/ml. The analyte showed excellent linear behavior over the specified range with coefficient of correlation (*R* ^2^) value of 1.

#### 3.2.3. Accuracy

The accuracy of the optimized method was determined by spiking the excipient samples with known amounts of formaldehyde at four concentration levels (*n* = 3). The results (shown in Table 1) were expressed as mean recoveries. The recovery at all concentration levels was within the acceptable range (80–120%). This indicates that the method is accurate.

#### 3.2.4. Precision

The precision of the method was evaluated in terms of the repeatability (expressed as intraday precision) and the intermediate precision (expressed as interday precision). The repeatability of the method was established from triplicate (*n* = 3) injections of each spiked excipient sample at each concentration level. Intermediate precision (interday precision) was carried out by analyzing the spiked excipient samples, prepared in the same way and at the same concentration levels, on two different days by two different analysts. The results of the method precision (shown in Table 3) were expressed as a relative standard deviation (RSD%). All RSDs were less than 4%. This indicates that the method is precise.

#### 3.2.5. Limits of Detection and Quantification

The limits of detection and quantification (LOD and LOQ, resp.) were evaluated based on signal-to-noise ratios of 3 and 10, respectively, and the respective values were found to be 121.80 and 406.00 *µ*g/L. Because the sample concentration was 50 mg/mL, LOD in excipient samples was 2.44 *µ*g/g, while LOQ was 8.12 *µ*g/g.

#### 3.2.6. Robustness

Changing the flow rate within ±0.1 mL/min had no effect on the area of formaldehyde derivative peak, and all RSDs were less than 5%. This indicates the robustness of the method.

### 3.3. Performance of the Proposed Method

Little analytical methods have been developed to determine formaldehyde in pharmaceutical excipients [13].  Table 4 shows a comparison between these methods and the proposed one.

Compared to other methods, the time required to complete formaldehyde determination using the developed method was generally faster. Recovery obtained with the presented procedure was comparable with those reported by others. Precision of the method was also close to those mentioned by others; however, it was better than that for SPME-based method. Although the proposed method had lower sensitivity than most of the other methods, however, it gives the opportunity to detect and determine formaldehyde impurity in pharmaceutical excipients below the limit stated in British pharmacopoeia (15 ppm for free formaldehyde in PEG) [5].

### 3.4. Analysis of Commercial Pharmaceutical Excipient Samples

PVP and PEG are widely used in many pharmaceutical applications and dosage forms. They are utilized in drug products over a wide range of concentrations. These concentrations can reach up to 67% for PEG 400 and 25% for various grades of PVP [3, 27].

The optimized SHS-GC-FID method was applied to analyze the presence of formaldehyde in several commercial PEG and PVP samples. The results of the analysis are summarized in Table 5.

Formaldehyde content in the excipient samples was extensively varied from 8.04 to 190.58 *µ*g/g. These variations depended on the nature, the storage conditions, and the source of the excipient samples.

Formaldehyde was not detected in PVP K-30 samples from the companies A and B. However, PVP samples (from the companies C and D) contained trace amount of formaldehyde. Actually, there is no specific pharmacopoeial limit for formaldehyde in PVP, but there is a general limit for aldehydes (500 ppm) which expressed as acetaldehyde [5, 6].

According to British Pharmacopoeia, the limit of formaldehyde in PEG is 15 ppm [5]. Both PEG 400 from the company E and PEG 300 from the company G had formaldehyde levels exceeding the identified limit.

Although PVP K-25 from the companies C and D and PEG 400 from the company F contained trace levels of formaldehyde, however, such levels may cause critical drug products instability and degradation, especially when excipient/active pharmaceutical ingredient ratio is high, according to many literatures [1, 2, 4].

## 4. Conclusion

A SHS-GC-FID method was developed and validated for determination of formaldehyde in pharmaceutical excipients. Samples were simply prepared in headspace vials by adding acidified ethanol as a diluent and derivatization reagent. After that, the vials were automatically moved to the incubator where formaldehyde derivative was simultaneously formed and extracted. The developed method was linear, specific, accurate, and precise over the specified range. The limits of detection and quantification were 2.44 and 8.12 *µ*g/g, respectively. The method was also simple and rapid, and it does not require sophisticated instrumentations or large amounts of solvents. This method was used to screen PEG and PVP samples for formaldehyde. The tested samples contained varying levels of it. So, the method could be valuable in selecting appropriate excipients and/or excipient batches for pharmaceutical formulation. It is also important for selecting approved vendors of pharmaceutical excipients. In addition, it can be applied as a tool for testing the quality of pharmaceutical excipients routinely.

## Figures and Tables

**Table 1:** Effect of incubation temperature on response (*n* = 2).

Incubation temperature (°C)	Response (mean ± SD)
60	230299 ± 4872
70	233366 ± 4779^a^

^a^No significant difference when compared with incubation at 60°C.

**Table 2:** Effect of water content on formaldehyde determination (*n* = 2).

Percentage of water relative to sample weight (%)	0	5	10	20
Formaldehyde concentration (*µ*g/mL) (mean ± SD)	1.45 ± 0.025	1.48 ± 0.009^a^	1.48 ± 0.025^a^	1.48 ± 0.015^a^

^a^No significant difference when compared with the control (0% water).

**Table 3:** Accuracy, intraday, and interday precision of the developed method.

Formaldehyde spiked (*µ*g/g)	PVP K-30			PEG 400		
	Mean recovery % (*n* = 3)	Intraday precision (RSD%) (*n* = 3)	Interday precision (RSD%) (*n* = 6)	Mean recovery % (*n* = 3)	Intraday precision (RSD%) (*n* = 3)	Interday precision (RSD%) (*n* = 6)
10.0	104.27	2.65	1.92	87.76	0.75	3.18
50.04	98.14	1.48	1.56	89.79	1.59	3.24
500.4	98.99	2.96	3.61	97.70	2.04	2.67
1000.9	97.89	1.52	1.56	101.12	2.07	2.05

**Table 4:** Comparison between the methods used to determine formaldehyde in excipients or drug substances.

Analytical technique	Derivatization and/or extraction times (min)	Run time (min)	Approx. recovery (%)	Approx. precision^a^ (%)	Approx. LOD^b^ (ppm)	Ref.
GC-FID	5	>7	No data	No data	7	[21]
GC-MS	240	15	86–99	3.7	0.02	[19]
SHS-GC-FID	30	5	85–97	≤3	0.05	[20]
SHS-GC-MS	20	28	No data	3.1	0.05	[20]
SPME-HS-GC-MS^c^	60	22.5	106.5–113.5	3.13–13.19	No data	[18]
HPLC-UV	No data	≥35	84–97	0.5–1.2	0.5	[22]
HPLC-UV	60	30	No data	No data	0.03	[15]
HPLC-UV	No data	No data	94.9–102.9	No data	10	[23]
Proposed method	15 or 25	11	87.8–104.3	0.75–3.6	2.44	—

^a^Including inter- and intraday precision; ^b^LOD in sample; ^c^SPME: solid-phase microextraction.

**Table 5:** Formaldehyde level in the excipient samples.

Company	Excipient	Formaldehyde level (*µ*g/g)
A	PVP K-30	ND^a^
B	PVP K-30	ND^a^
C	PVP K-30	3.51^b^
D	PVP K-25	8.04
E	PEG 400	22.47
F	PEG 400	3.64^b^
G	PEG 300	190.58

^a^Not detected; ^b^formaldehyde level < QL.

## Supplementary Materials

Supplementary 1 Figure S1: representative GC-FID chromatograms of specificity study.

Supplementary 2 Figure S2: GC-FID chromatogram of PEG 400 sample spiked with formaldehyde. DEM: diethoxymethane.

Supplementary 3 Figure S3: GC-FID chromatogram PVP K-30 sample spiked with formaldehyde. DEM: diethoxymethane.
