# Supplementary material for: A Robust Static Headspace GC-FID Method to Detect and Quantify Formaldehyde Impurity in Pharmaceutical Excipients
Source: J Anal Methods Chem. 2018 Mar 4;2018:4526396. doi: 10.1155/2018/4526396 (PMC5857322; doi:10.1155/2018/4526396)
Supplement: Supplementary 1 — Figure S1: representative GC-FID chromatograms of specificity study. [file 4526396.f1.docx]

Fig. S1 without labels.


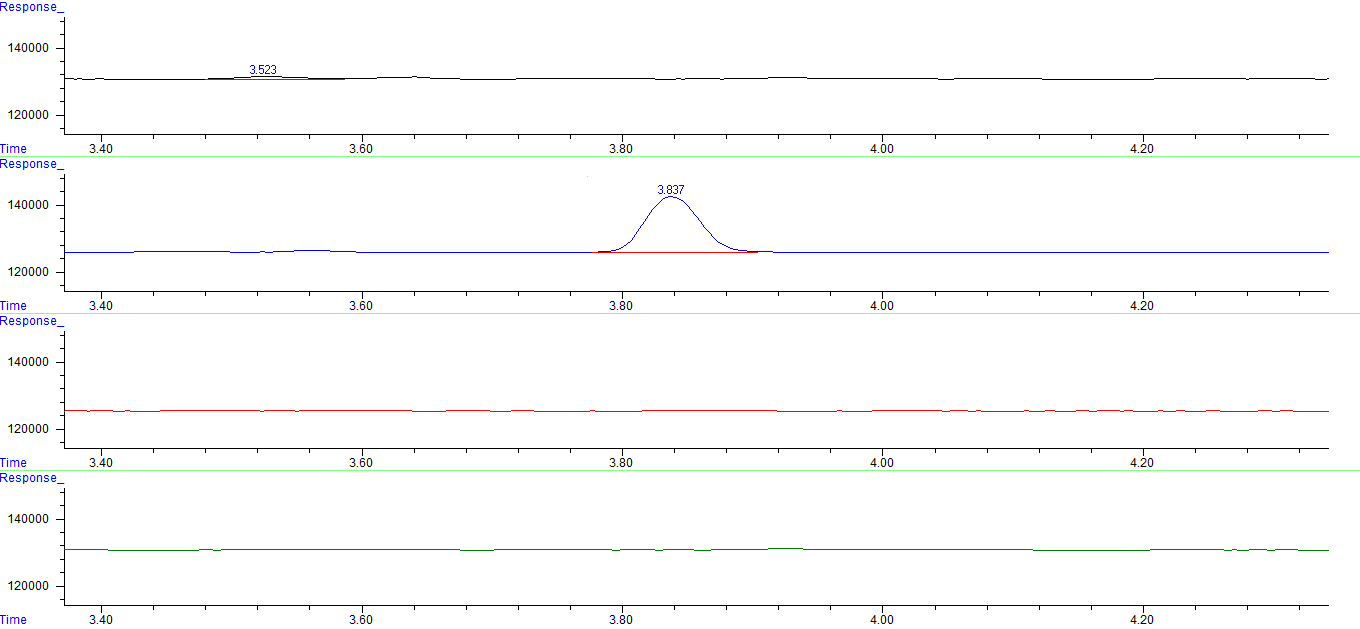


Fig. S1 with labels.


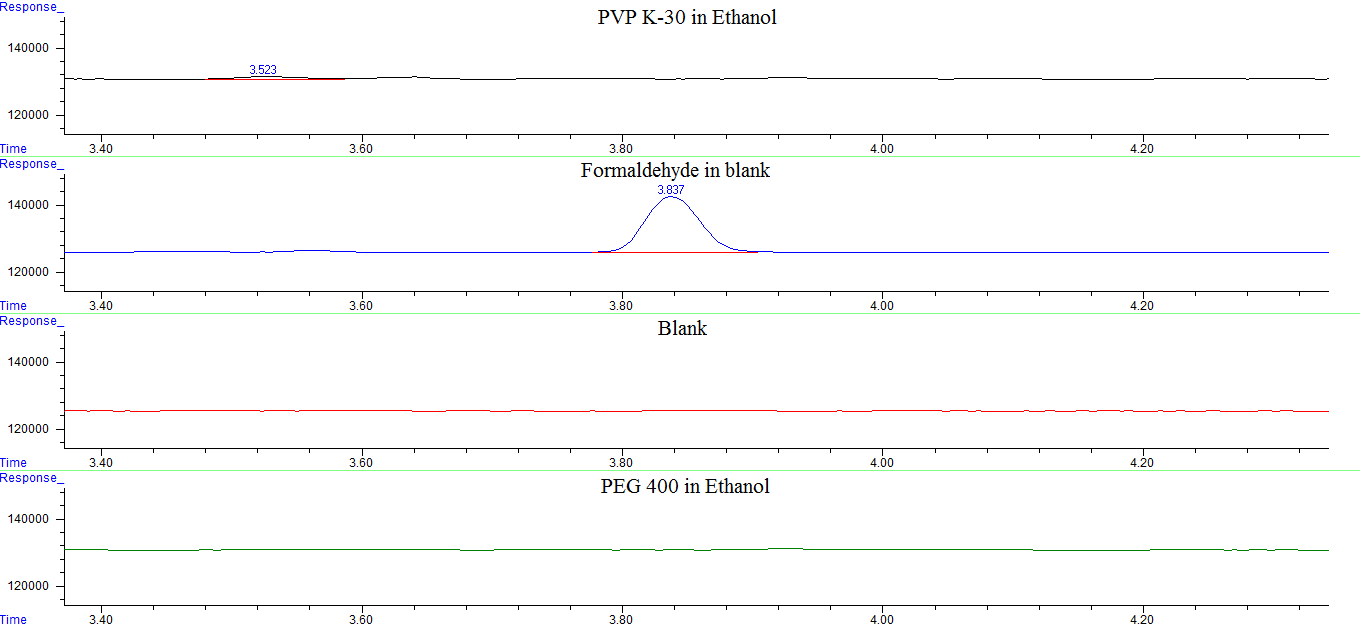


Figure S1. Representative GC-FID chromatograms of specificity study.
