# Supplementary material for: A Robust Static Headspace GC-FID Method to Detect and Quantify Formaldehyde Impurity in Pharmaceutical Excipients
Source: J Anal Methods Chem. 2018 Mar 4;2018:4526396. doi: 10.1155/2018/4526396 (PMC5857322; doi:10.1155/2018/4526396)
Supplement: Supplementary 2 — Figure S2: GC-FID chromatogram of PEG 400 sample spiked with formaldehyde. DEM: diethoxymethane. [file 4526396.f2.docx]

Fig. S2 without labels.


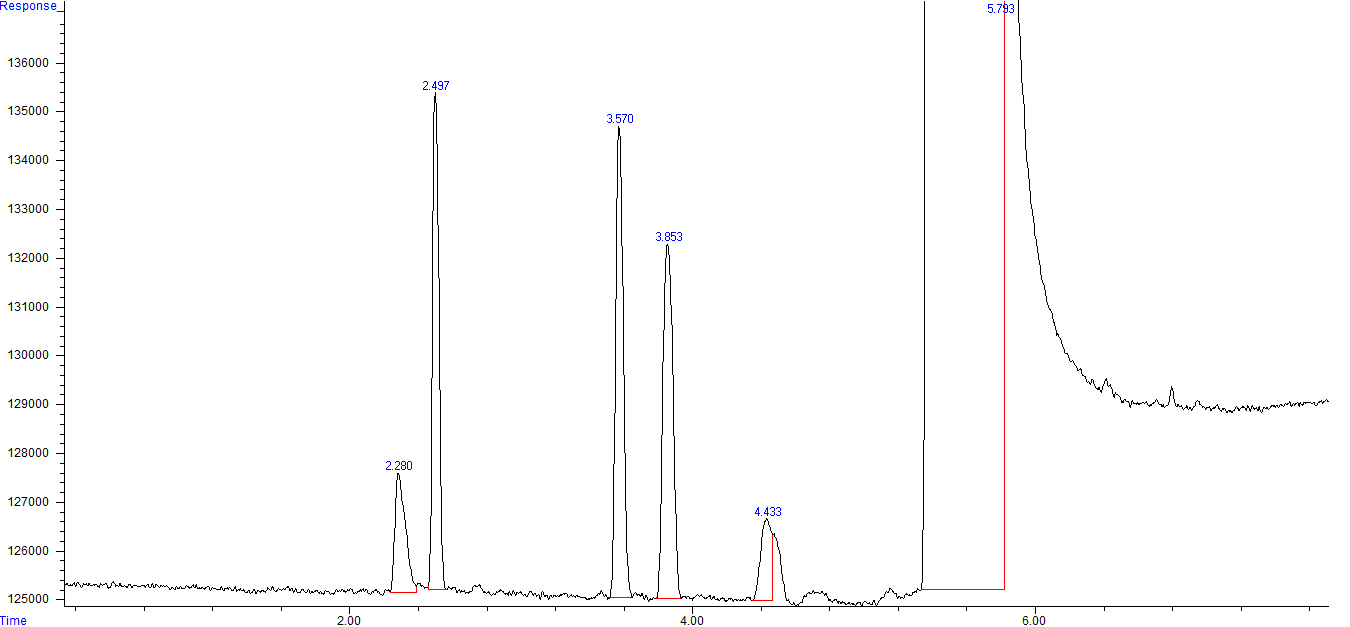


Fig. S2 with labels.


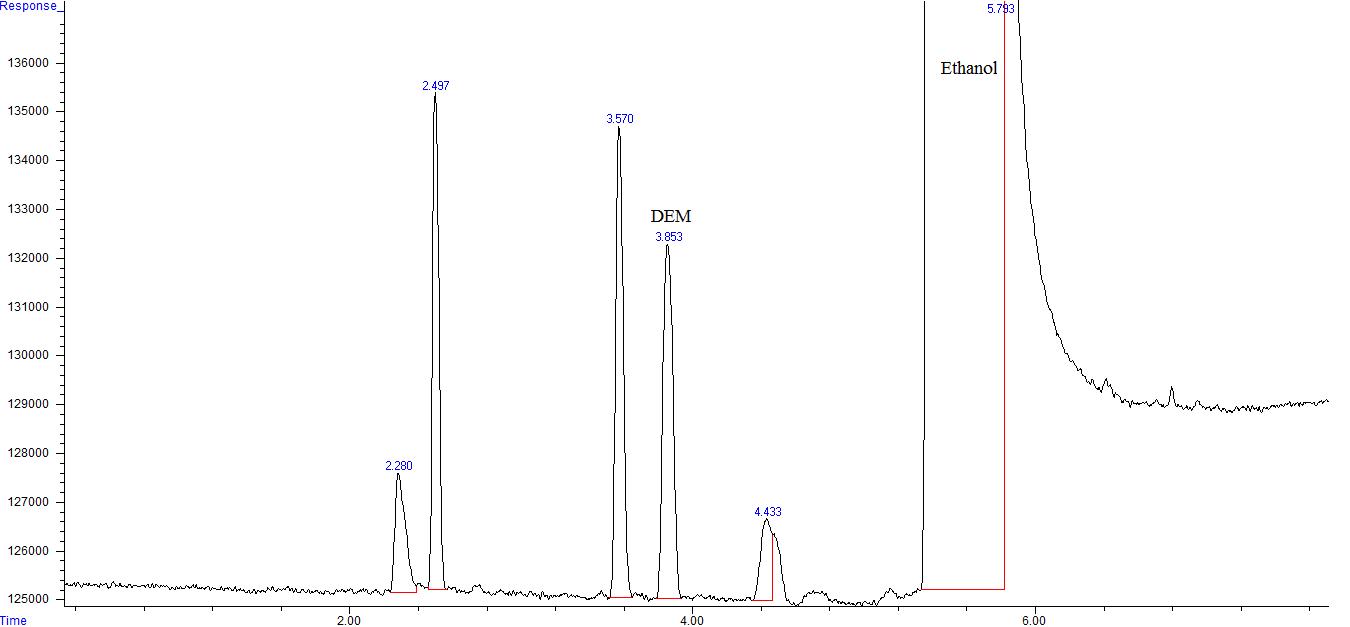


Figure S2. GC-FID chromatogram of PEG 400 sample spiked with formaldehyde. DEM: diethoxymethane.
